# Effects of cerebellar transcranial alternating current stimulation in cerebellar ataxia: study protocol for a randomised controlled trial

**DOI:** 10.3389/fnins.2023.1180454

**Published:** 2023-04-27

**Authors:** Xia Liu, Wei Lin, Lin Zhang, Wan-Li Zhang, Xiao-Ping Cheng, Yan-Hua Lian, Meng-Cheng Li, Shi-Zhong Wang, Xin-Yuan Chen, Shi-Rui Gan

**Affiliations:** ^1^Department of Rehabilitation Medicine, The First Affiliated Hospital, Fujian Medical University, Fuzhou, China; ^2^Department of Neurology and Institute of Neurology, The First Affiliated Hospital, Fujian Medical University, Fuzhou, China; ^3^Department of Radiology, The First Affiliated Hospital, Fujian Medical University, Fuzhou, China; ^4^College of Mechanical Engineering and Automation, Fuzhou University, Fuzhou, China; ^5^The School of Health, Fujian Medical University, Fuzhou, China; ^6^National Regional Medical Center, Binhai Campus of the First Affiliated Hospital, Fujian Medical University, Fuzhou, China

**Keywords:** spinocerebellar ataxia, multiple system atrophy, transcranial alternating current stimulation, randomised controlled trial, treatment

## Abstract

**Background:**

Cerebellar ataxia (CA) is a movement disorder that can affect balance and gait, limb movement, oculomotor control, and cognition. Multiple system atrophy-cerebellar type (MSA-C) and spinocerebellar ataxia type 3 (SCA3) are the most common forms of CA, for which no effective treatment is currently available. Transcranial alternating current stimulation (tACS) is a non-invasive method of brain stimulation supposed to alter cortical excitability and brain electrical activity, modulating functional connectivity within the brain. The cerebellar tACS can modulate the cerebellar outflow and cerebellum-linked behavior and it is a proven safe technique for humans. Therefore, the aim of this study is to 1) examine whether cerebellar tACS improves ataxia severity and various non-motor symptoms in a homogeneous cohort of CA patients consisting of MSA-C and SCA3, 2) explore the time course of these effects, and 3) assess the safety and tolerance of cerebellar tACS in all participants.

**Methods/design:**

This is a 2-week, triple-blind, randomised, sham-controlled study. 164 patients (MSA-C: 84, SCA3: 80) will be recruited and randomly assigned to either active cerebellar tACS or sham cerebellar tACS, in a 1:1 ratio. Patients, investigators, and outcome assessors are unaware of treatment allocation. Cerebellar tACS (40 min, 2 mA, ramp-up and down periods of 10s each) will be delivered over 10 sessions, distributed in two groups of five consecutive days with a two-day break in between. Outcomes are assessed after the tenth stimulation (T1), and after 1 month (T2) and 3 months (T3). The primary outcome measure is the difference between the active and sham groups in the proportion of patients with an improvement of 1.5 points in the Scale for the Assessment and Rating of Ataxia (SARA) score after 2 weeks of treatment. In addition, effects on a variety of non-motor symptoms, quality of life, and autonomic nerve dysfunctions are assessed via relative scales. Gait imbalance, dysarthria, and finger dexterity are objectively valued via relative tools. Finally, functional magnetic resonance imaging is performed to explore the possible mechanism of treatment effects.

**Discussion:**

The results of this study will inform whether repeated sessions of active cerebellar tACS benefit CA patients and whether this form of non-invasive stimulation might be a novel therapeutic approach to consider in a neuro-rehabilitation setting.

**Clinical Trial Registration**: ClinicalTrials.gov, identifier NCT05557786; https://www.clinicaltrials.gov/ct2/show/NCT05557786.

## Introduction

Cerebellar ataxia (CA) is a major cause of gait imbalance, limb dyskinesia, impaired motor-ocular control, and cognitive impairment (Manto et al., 2020). CA can be divided into sporadic ataxia and inherited ataxia, with the multiple system atrophy of cerebellar type (MSA-C) and spinocerebellar ataxia type 3 (SCA3) as the most common types, respectively (Anheim et al., 2012; Poewe et al., 2022). Although the pathogenic factors of these two types of CA are complex and varied, the common pathological features are injury, atrophy or dysfunction of the cerebellar and/or its afferent/efferent neural pathways (Radmard et al., 2023). At present, except for a few CA with very clear pathogenesis, there is still a lack of effective targeted treatment in clinic (Marmolino and Manto, 2010; Gandini et al., 2020; Beaudin et al., 2022; Correia et al., 2023). Therefore, an in-depth study of the neural mechanism of CA is of great significance for understanding cerebellar motor regulation and developing new therapeutic targets and strategies.

Transcranial alternating current stimulation (tACS) delivers brain stimulation by modulating cortical excitability and spontaneous brain activity in the scalp via a weak electrical current (Cabral-Calderin and Wilke, 2020). In the context of human neuroscience research, cerebellar tACS (CB-tACS) technique was pioneered by Mehta and colleagues (Mehta et al., 2014). This technique facilitates the study of cerebellar oscillations through the interaction of reactive neuronal elements (Wessel et al., 2022), enriches the imaging method of electrophysiology, and affects corticospinal excitability through the thalamic cortical pathway of the cerebellum, helping to explore the oscillatory mechanism triggered by the cerebellum and its associated circuits (Asan and Sahin, 2019; Wessel et al., 2022). A growing number of studies have demonstrated the ability of tACS to modulate different domains of human behavior rhythm and gait (Koganemaru et al., 2020), such as motor learning (Schubert et al., 2021), improving motor skills (Wessel et al., 2020), working memory (Abellaneda-Pérez et al., 2019; Grover et al., 2022), and the processing of emotional stimuli (Hu et al., 2021) and cognition (Del Felice et al., 2019). Furthermore, the safety of CB-tACS has been confirmed in healthy adult studies with only a few subjects reporting scalp burning, mild tingling, phosphorescent sensation, and other transient adverse reactions (Antal et al., 2017; Matsumoto and Ugawa, 2017). More recently, research has also begun to explore its therapeutic potential in various neurological disorders. Although tACS has been suggested as a treatment for different neurological conditions (PD (Del Felice et al., 2019), AD (Benussi et al., 2022), schizophrenia (Ahn et al., 2019), depression (Wang et al., 2022), and insomnia (Hong-Xing et al., 2020)), no evidence was reported yet for the tACS treatment on CA patients.

Based on these abovementioned results, we performed our clinical CB-tACS study. Our aims were 1) investigate whether cerebellar tACS decreases ataxia severity and a variety of non-motor symptoms in a homogeneous cohort of patients of MSA and SCA3 and 2) what is the duration of this beneficial effect.

## Methods

### Ethics and dissemination

This study was carried out according to the Declaration of Helsinki and got the approval of the local ethics committee of The First Affiliated Hospital of Fujian Medical University, Fuzhou, China (MRCTA, ECFAH of FMU [2022]399) on Aug 5, 2022. The contacts of the ethics committee are: 0086–0591-87,981,028, fykyll@163.com, and No.20, Chazhong Road, Fuzhou, Fujian Province, China. Participants need to sign an informed consent form before this study and receive medical care after the study. The research results will be published through articles or conferences. Thus, this study will benefit the treatment of CA patients and hopefully supply an effective non-pharmacological intervention soon.

### Experimental design, randomization, and blinding

**Figure 1:**
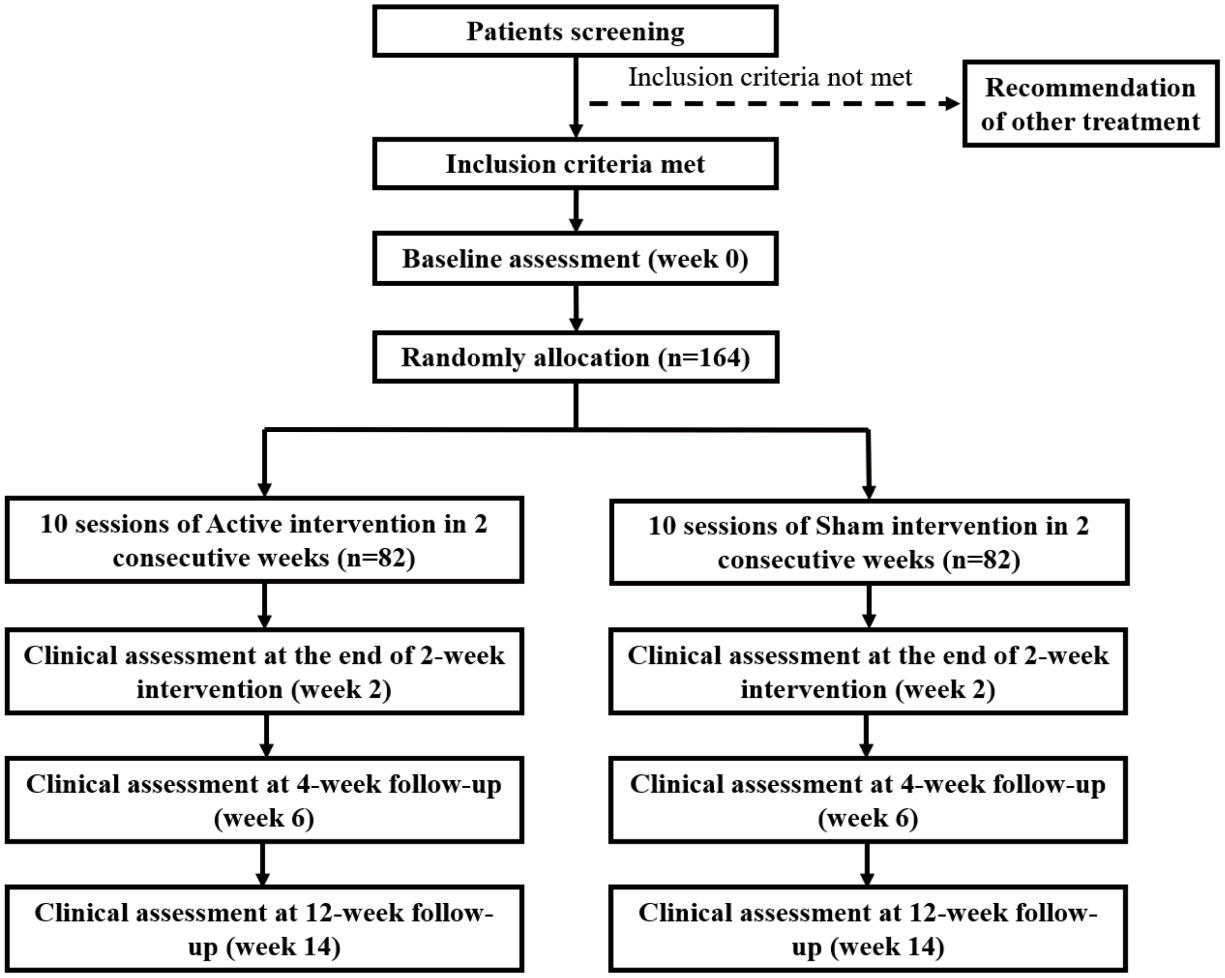
Design and flow of participants through the study.

This is a single-centre, triple-blind, randomised, sham-controlled intervention trial. The total of 164 recruited participants with CA (MSA: 84, SCA3: 80) will be randomly and equally assigned to the active or sham treatment group. The randomization was created by a computer program in permuted blocks of four and managed by an offsite statistician who does not participate in this study. Every participant gets an unknown-beforehand number from a sealed opaque envelope, which determines the trial he/she will participate in. The envelopes for the two tACS plans (sham and active) have the same characteristics regarding size, color, appearance, weight, and odor, and different plans are numbered by the statistician. All participants were blinded to the specific assignment during the triple-blind treatment period until the end of the follow-up, except for any emergency when the blinding will be stopped. Participants will receive a 2-week intervention, followed by a 4-week follow-up (conducted at the 6th week) and a 12-week follow-up (conducted at the 14th week). The entire process will strictly follow the consolidated standards of reporting trials guidelines (Figure 1) and standard protocol items: recommendations for interventional trials checklist (Chan et al., 2013a, b).

Termination of the trial will be decided by the principal investigators, according to the following criteria: (1) lack of two consecutive tACS sessions, (2) severe adverse events (AEs), and (3) affected tACS-Assessment by treatment of other diseases. If a patient is lost to follow-up at weeks 6 or 14, the data collected up to that point will still be used in the statistical analysis at T2/T3.

### Study participants

Participants with CA who will receive research at the clinical Observation Cohort study of CA (NCT04010214) in the Department of Neurology, the First Affiliated Hospital of Fujian Medical University from August 2022 to April 2023 by our investigators will be identified for their eligibilities. All eligible participants will get access to the specific treatment plans and they can register directly to attend the trial for randomization. All potential risks will be informed in the consent. The withdrawal reason will be carefully recorded if any participant quits halfway. To maximize trial compliance, potential risks, requirements, the study schedule, and benefits will be fully explained to all recruited participants.

Participants will be instructed to avoid any anti-rehabilitation treatment for CA during the study. All participants will have freedom to quit at any time and choose other medical therapy strategies.

## Eligibility criteria

### Inclusion criteria

- 1 Inclusion Criteria for SCA3 patients:
- 1.1 Detectable clinical symptoms and a confirmed diagnosis for SCA3.
- 1.2 18–80 years old.
- 1.3 Signed informed consent by patients or their family members.
- 1.4 3-30pre-study Scale for the Assessment and Rating of Ataxia (SARA).
- 2.Inclusion Criteria for MSA-C:
- 2.1 30–80 years old;
- 2.2 Clinically diagnose or probable MSA-C according to the latest MSA diagnostic criteria (Wenning et al., 2022);
- 2.3 <4 years MSA-C medical history;
- 2.4 Independent walk (or with assistance);
- 2.5 >3 years life expectancy;
- 2.6 Contraceptive measures for women of childbearing age.
- 3. Exclusion Criteria for both SCA3 and MSA-C:
- 3.1 Patients with medical history of stroke, encephalitis, and epilepsy.
- 3.2 Patients with serious cognitive and behavioral disorders, or mental illness.
- 3.3 Patients with severe medical illness (such as kidney failure, convulsions, stomach ulcers, liver disease) and uncontrolled high blood pressure or diabetes.
- 3.4 Patients with head injury, neurosurgery, or mental issues within the head.
- 3.5 Patients who took investigational products within 4 weeks prior to this enrollment, or who are pregnant or breastfeeding.
- 3.6 Patients with metallic particles in the eye, medical pumps like implanted cardiac pacemaker or neurostimulators, and surgical clips (above the shoulder line), etc.

### Sample size calculation

Positive effect is supposed to occur in both groups according to previous report (Romano et al., 2015). The primary criterion was a ≥ 1.5 points SARA score decrease caused by 2 weeks of treatment. The SARA scores was expected to increase in 25% of placebo patients and 60% of the treated patients. 80% power was tested for 36 patients in each group, with a 35% δ between the groups, by using a bilateral test and 5% *α*. Taking 10% attrition rate in consideration, 40 patients for each group will be needed to reach a minimum total sample size of 72. Based on these calculations, we estimate that 80 SCA3 participants will enroll in the study.

Our sample size was calculated as previously described (Levin et al., 2019). This study is expected to produce *α* of 0.05 at 80% power for a treatment effect of 50% on the annual progression of the movement examination score on the Unified Multiple System Atrophy Rating Scale (UMSARS); this contains 4 points from the active group and 8 points from the placebo group, which is above the minimal clinically detectable significant level of 3.8 points (Krismer et al., 2016). Therefore, we conducted this study to determine whether active tACS treatment would have a positive effect on MSA-C patients. The target total patients was 84, with an expected attrition rate of 10%.

### TACS intervention

Professional doctors will guide the participants to accept Active tACS (A-tACS) or Sham tACS (S-tACS) intervention under the same conditions during all sessions for 2 weeks. The tACS session was delivered via three 0.9% NaCl soaked surface sponge electrodes (5X7cm) on a current stimulator (Neustim NSS18, Neuracle, Changzhou, China). The return electrode was placed 2 cm below the inion and the two active electrodes were over the bilateral buccinator muscles. After computational modeling of electric field distribution, we chose this tACS particular montage with an extracephalic electrode, which can lead to significant entrainment of brain oscillations (Sadeghihassanabadi et al., 2022). Electrodes are fixed with elastic gauze and coated with conductive gel to reduce contact resistance (<5 kΩ).

An alternating sinusoidal current of 1 mA peak-to-baseline with a frequency of 70 Hz was used to stimulate the A-tACS throughout the whole behavioral treatment session (40 min) based on previous studies (Hong-Xing et al., 2020; Wang et al., 2022). The molecular mechanism of tACS remains unclear (Wessel et al., 2022). Currently, *γ*-tACS is most commonly applied into neurodegeneration and psychiatric disorders, γ-band is about 30 ~ 80 Hz, the electrical activity of the brain is mainly involved in cognitive function (Hu et al., 2021; Grover et al., 2022), motor function (Wessel et al., 2020; Giustiniani et al., 2021; Miyaguchi et al., 2022), and abnormal γ activity is commonly seen in various neuropsychiatric diseases. Therefore, gamma waves were selected in our study to explore the efficacy in cerebellar ataxia. For the S-tACS stimulation, the electrode placement was as the same as A-tACS, but the current was gradually decreased 40s after stimulus onset to simulate the experimental stimulus; in this manner, patients were blinded whether they were treated with active or sham tACS.

Participants were asked to sit in a bright and quiet room during all stimulation period, with their eyes open but no speaking and no large movement. Each participant will receive 10 interventions within 2 weeks, once a day from Monday to Friday. To examine differences between simulated perceptions, participants need to answer whether they feel they were treated with real or sham stimulation, and whether they experienced tingling skin sensations or phosphenes/light flickers. Sensations were rated from 0 (no sensation) to 4 (very strong sensations). The treatment type (A-tACS vs. S-tACS) was encoded in the in-house software and was masked for both patients and the researchers. Researchers only needed to enter a patient name and session number to start stimulation, but the technician confirmed whether an active or sham stimulation was delivered after each treatment was done.

### Outcome measures

Considering the wide clinical signs and symptoms of CA, various outcome measures which were validated for CA were selected to determine the improvement degrees of motor and non-motor functions. Measurements will be carried out under supervision, and data will be collected by specialist examiners or trained doctors. All results were obtained at baseline, weeks 2, 6, and 14, respectively (Table 1).

**Table 1:** Schedule of enrolment, interventions, and assessments.

Week	Screening-2w	Baseline (T0) 0w	Treatment (T1) 2w	Follow-up (T2) 6w	Follow-up (T3) 14w
Enrolment					
Signed informed consent		√			
Diagnosis	√				
Randomization		√			
Interventions					
Sham intervention group		√	√		
Active intervention group		√	√		
Assessments					
Primary outcome					
SARA		√	√	√	√
UMSARS (only for MSA-C)		√	√	√	√
Secondary outcomes					
ICARS		√	√	√	√
9HPT		√	√	√	√
Dysarthria		√	√	√	√
Fatigue-14		√	√	√	√
PSQI		√	√	√	√
Epworth score		√	√	√	√
MoCA		√	√	√	√
MMSE		√	√	√	√
HAMA		√	√	√	√
HAMD		√	√	√	√
EQ-5D-5L/MSA-QoL		√	√	√	√
SCOPA-Aut(only for MSA-C)		√	√	√	√
Gait parameters		√	√	√	√
EEG		√	√	√	√
fMRI		√	√	√	√
Temperature, blood pressure, heart rate	√		√	√	√
Patients’ compliance		√	√	√	√
Blinding assessment		√	√	√	√

### Primary outcome measurements

The primary outcome for both SCA3 and MSA-C was the difference between the active and placebo groups in the proportion of patients with an improvement of 1.5 points in SARA score after 2 weeks of treatment. SARA (Schmitz-Hübsch et al., 2006) consists of 8 items: sitting, gait, finger chase, stance, speech disturbance, fast alternating hand movements, heel-shin slide, and nose-finger test. Higher score represents worse performance. SARA scoring will be done by experienced but blinded investigators under videotaping.

There was an alternative primary outcome for MSA-C, which was the difference in total UMSARS score between the two groups being treated for 2 weeks. The UMSARS consists of four parts (I, II, III, and IV) (Wenning et al., 2004), in which UMSARS-I and II are usually taken as key endpoints during clinical trials (Palma et al., 2022).

### Secondary outcome measurements

Except for the cardinal symptom of ataxia, there are a variety of complex non-motor symptoms in SCA3 and MSA-C, including cognitive and emotional impairments, fatigue, sleep disorders, and dysfunction of the autonomic nerve. Therefore, in order to comprehensively analyze the treatment effect and its duration, we further studied the variation of a series of motor and non-motor indicators as the secondary outcome measurements at the end of the treatment and at 1 and 3 months after the treatment.

### Assessment of symptom of ataxia

To further investigate the treatment effect in ataxia, we adopted ICARS, which is a widely used scale for the ataxia severity measurement. The ICARS is composed by 19 items from four subscales, including 7 posture and gait disturbances, 7 kinetic functions, 2 speech disorders, and 3 oculomotor disorders. The total 0–100 scores allow for individual subscore’s analysis (Trouillas et al., 1997).

### Assessment of non-motor symptoms

To investigate whether the treatment improves the non-motor symptoms, the following scales are used: The Montreal Cognitive Assessment (MoCA) (Nasreddine et al., 2005) and The Mini-Mental State Examination (MMSE) (Folstein et al., 1975) for evaluating cognitive change; Hamilton Anxiety Scale (HAMA) (HAMILTON Hamilton, 1959) and Hamilton Rating Scale for Depression (HAMD-17) (Lin et al., 2018) for evaluating the changes in the status of anxiety and depression, respectively; The 14-item Fatigue Scale (FS-14) (Chalder et al., 1993) for evaluating symptoms of fatigue; finally, Sleep habits self-assessment (Pittsburgh Sleep Quality Index (PSQI) (Buysse et al., 1989) and Epworth Sleepiness Scale (ESS) (Johns, 1991) are used for investigating the improvement in sleep disturbance.

### Assessment of life ability and quality of life

To explore whether the treatment improves life quality of patients, the EuroQol Five-dimensional questionnaire (EQ-5D) and the Multiple System Atrophy Quality of Life questionnaires (MSA-QoL) are used for the participants of SCA3 and MSA-C, respectively. EQ-5D is an ordinal quality of life scale that can address the problems from mobility, selfcare, usual activities, pain/discomfort, or anxiety/depression (Bolzan et al., 2022). MSA-QoL is a MSA-specific self-report assessment. MSA-QoL and UMSARS have the same motor subscales (Schrag et al., 2007).

### Assessment of dysfunction of autonomic nerve

To explore whether the treatment improves the symptoms of dysfunction of the autonomic nerve in participants of MSA-C, the SCOPA-Aut questionnaire (0–69) is assessed (Visser et al., 2004). The scores of 26 SCOPA-Aut items range from 0 to 69, with higher scores indicating more severe symptoms.

### Assessment of finger dexterity

The 9-Hole Peg Test (9HPT) were performed under limited time to examine the finger dexterity and upper limb coordination as previously described (Feys et al., 2017).

### Assessment of dysarthria

We used the speech sounds to analyze the following parameters through the DIVAS2.5 voice analysis system (Wuyts et al., 2000): (1) Fundamental frequency perturbation (Jitter) and amplitude perturbation (Shimmer) reflect the stability of vocal cord vibration, which is related to the degree of roughness and hoarse sound, (2) Intensity (including maximum intensity (SPLmax)), minimum intensity (SPLmin) and range of intensity (SPLrng), reflects the maximum, minimum and range of sound intensity during phonation, and is related to the closure degree of the glottis, (3) The longest articulation time (MPT) assesses respiratory function and glottic closure, and (4) Dysphonia severity index (DSI) is a comprehensive evaluation parameter of pronunciation.

### Assessment of gait imbalance

To objectively and quantitatively evaluate the improvement of gait after the treatment, we used a gait analysis system, which consists of a gait acquisition system and gait analysis software. The gait acquisition system consists of two smart insoles (20 sensing points) and two nine-axis gyroscopes. The smart insoles are placed in the shoes for the left and right feet, and the gyroscopes are placed on the toes of the left and right feet, respectively.

The subjects wore the gait acquisition system and completed three tasks at designated locations: (1) 3 min-standing with eyes open, (2) 3 min-standing with eyes closed, and (3) walking in the corridor at a normal speed for 20 m. A series of gait parameters are then processed and calculated in gait analysis software: (1) Gait speed (meters walked per second), (2) Step length (distance between left heel and right foot contact point) (in meters), (3) Cadence (steps per minute), (4) Gait cycle (the time from when one heel hits the ground to when the heel hits the ground again) (in seconds), (5) Single support phase (percentage of the gait cycle from the time when one heel hits the ground to the time when the toe leaves the ground on this side), (6) Double support phase (percentage of gait cycle during which one side is single support period and the other side is also single support period), (7) Toe-out angle (the angle between one side of the foot and the walking direction during the walking phase) (in degree), (8) Toe-off angle (the angle when one toe leaves the ground) (in degree), (9) Average value of plantar pressure (in N), (10) Variance of plantar pressure, (11) Variation coefficient of step length (step length average/standard deviation), and (12) Variation coefficient of gait cycle (gait cycle average/standard deviation).

### Assessment of neuroimaging characters

To explore the possible mechanism of the treatment, we performed Resting-state functional magnetic resonance imaging (RS-fMRI), which can reflect neural activity through blood oxygen level-dependent (BOLD) signals and be used to analyze functional changes of the brain. There are three commonly used indicators for RS-fMRI: (1) Amplitude of low frequency (ALFF) indicates the intensity of brain activity in a specific region and (2) ReHo is an indicator for the synchronization of neuronal activity (Zang et al., 2004); Functional connectivity (FC) represents the synchronization of different neurophysiological events with spatial distance (Friston et al., 1993), identifies reliable patterns of covarying brain signals that indicate neural activity.

For the magnetic resonance image acquisition and data preprocessing, all participants used the same neuroimaging process via a Skyra scanner (3.0 Tesla Siemens) with a 20-channel head and neck coil, in which a head holder is set to minimize the head movement. Sagittal anatomical images were obtained using T1-weighted three-dimensional (3D) magnetization prepared rapid gradient echo (MP-RAGE) sequences with the following scan parameters: Repetition time (TR) = 2,300 ms, echo time (TE) = 2.3 ms, reversal time (TI) = 900 ms, flip Angle = 8°, field of view (FOV) = 240 mm × 256 mm, matrix size = 240 × 256, Bandwidth = 200 Hz/Px, slice = 192, voxel size = 1 mm × 1 mm × 1 mm, total acquisition time = 5 min and 12 s. All MRI data were quality-controlled by an experienced radiologist.

The data of resting-state functional Magnetic Resonance Imaging (RS-fMRI) were pre-processed by using MATLAB R2016b-DPARSFA. The image preprocessing process is as follows: (1) The first ten functional images were discarded for signal equilibration, (2) The remaining volumes were co-registered to the individual’s corresponding MPRAGE image after the slice-timing correction and spatial realignme, (3) The anatomical images of gray matter, white matter (WM), and cerebrospinal fluid (CSF) were normalized to a 3x3x3mm3 space according to the Montreal Neurological Institute (MNI) standard, (4) The WM-and CSF-extracted average signals and the head movements estimated from the Friston 24-parameter model were used for physiological noise removal by nuisance regression, and (5) Detrend and bandpass filtering (0.01–0.09 Hz).

The amplitude of low frequency (ALFF) value was calculated as described (Kiviniemi et al., 2004). After subtracting the mean, ALFF was divided by the whole-brain voxel bias, followed by the normalization to make a Z-distribution, and the bandpass filtering.

The similarity between a single voxel and its surrounding 27 voxels was analyzed based on Regional homogeneity (ReHo) using Kendall’s coefficient of consistency (KCC). The individual ReHo value was divided by the average ReHo value of all groups. And finally, a 6-mm smoothing check was used to smoothen the ReHo brain map spatially (Zang et al., 2004).

The connections between seed points and the whole brain were examined by FC. If there is a statistical relationship between the time series of two regions, it can be assumed that the functional behavior of the two regions is correlated and that they are coupled to each other or are components of the same network. First, a seed region of interest was mapped out, and second, time series was extracted to perform Pearson correlation analysis with other regions; the goal was to examine whether the activity patterns of other brain regions were time-related to the activity patterns observed in the seed region.

### Safety variables

Vital signs will be examined at baseline, week 2, 6, and 14, respectively. All AEs collected will be recorded in the case report form (CRF) and their duration, severity, development, and causal relationship to the tACS will be evaluated. Once serious AEs occur (such as hospitalization, risk of death, significant or persistent disability, or incapacity), their details should be recorded in CRF, reported to the local ethics committee, principal investigators, and the China FDA within 24 h, and tracked until their disappearance or losing clinical significance. In addition, the adverse reactions during the treatments will be recorded by the self-made questionnaire (Wang et al., 2022) consisting of 18 items will be used to evaluate whether each in our study participant has the common tACS-associated AEs, such as adverse reactions.

### Data processing and quality control

To ensure the rigor of this study, the whole process will be carried out strictly according to Guidelines for Good Clinical Practice of the International Conference on Harmonisation (ICH). Data will be collected from baseline, week 2, 6, and 14, respectively (Table 1). All investigators, trial supervisors, and raters involved in the study must be trained before they participate in communicating and instructing participants, assessing, collecting data, and completing the CRF. Double entries of data into the software DeZhentech EDV V1.0 (Dezhen, China) will be finished by two independent investigators, and these electronic data will be stored on a secure university server with regularly back-up and password protection. DeZhentech EDV V1.0 has an authority management mechanism associated with personnel and roles and a strict data audit mechanism to fully protect the security of data utilisation. Double-check will be performed to correct inconsistent entries or typos. The details of the quit participants, including reasons, date, AEs, and duration of the treatment, will be recorded. The final trial dataset, including the intent-to-treat and per-protocol dataset will be analyzed by an independent statistician.

### Statistical analysis

SPSS statistical software (version 26.0, United States) was used for statistical analysis. *p* < 0.05 represents significant difference. All tests are two-sided. Mean and standard deviation will be used for continuous variables, and frequency and percentage will be used to represent categorical variables. Regarding the comparison between groups, the continuous and categorical variables between groups were tested by Mann–Whitney U and the Chi-square test (or Fisher exact test), respectively. The intention-to-treat analysis works for the primary outcome, with worst-case imputation and repeated continuous outcomes represented by a mixed linear model.

## Discussion

The therapeutic effects of a cerebellar tACS in CA patients will be evaluated by our designed trial which is randomised, double-blind, and sham-controlled. We observed that patients with less severe ataxia showed the largest decrease, which is consistent with a previous study (Benussi et al., 2017) and suggests the regulation of motor activity by the volume of viable cerebellar cortex via the cerebello-thalamocortical connections. For this reason, our study will include individuals who were affected by CA mildly to moderately. In this trial, we mainly aim to investigate the effects of cerebellar tACS on CA severity, we also display the defective spectrum in SCA3 patients by using various outcome measures.

TACS is a common non-invasive form of brain stimulation (Antal et al., 2008; Bologna et al., 2019), which transfers low-intensity sinusoidal alternating current to the scalp and regulates its internal nerve oscillation (Elyamany et al., 2021) by forcing the resting membrane potential to a slightly-increased depolarisation or hyperpolarisation (Wu et al., 2021). In the depolarisation state, stochastic resonance occurs (Fertonani and Miniussi, 2017), and it locks the firing time of neurons to the increased stimulation frequency (Del Felice et al., 2019). Therefore, tACS can regulate (but not dominant) the causal relationship between neural activity and behavior (Herrmann et al., 2013).

Compared with the direct current stimulation of tDCS, tACS delivers current in a bidirectional manner (Kim et al., 2021). The advantage of tACS is its ability to manipulate and modulate inherent brain oscillations by inputting sinusoidal, biphasic alternating current (Yavari et al., 2018). In some frequency ranges, tACS treatment can induce endogenous brain oscillations, and when the amplitude of this stimulation increases, it causes the brain to oscillate over a wider frequency range (Ali et al., 2013). The tACS, whose current phases alternate regularly between positive and negative voltages, have been shown to be more effective than tDCS in regulating brain oscillations (Ali et al., 2013). Another advantage of tACS is that it can completely bypass sensory stimuli and induce endogenous oscillations through an external, barely perceptible alternating current, in which the endogenous oscillations are synchronized with the exogenous, rhythmic stimuli (Herrmann et al., 2013). So we want to bring this potential treatment to CA patients.

Although we obtained some valuable data from this trial and they will provide non-pharmacological interventions to treat ataxia severity with minimal side effects, but this trial also has some limitations. Firstly, the study is performed in a single centre, thus it may not represent the results from other regions (The Han nationality is the majority in China). Secondly, the intervention duration may not be long enough and it is uncertain how many interventions will bring the best effect. Thirdly, still have some to-be-solved questions, including whether (and to what extend) other characteristically differences in patients may influence outcomes (Fava et al., 2017). We will perform further researches to improve the outcomes of this trial in the future.

## Ethics statement

The studies involving human participants were reviewed and approved by MRCTA, ECFAH of FMU [2022]399. The patients/participants provided their written informed consent to participate in this study.

## Author contributions

XL: designed the study and drafted the manuscript. WL, LZ,W-LZ, X-PC, Y-HL, and M-CL: collected the clinical data. S-RG, S-ZW, and X-YC: critically revised the manuscript and contributed the most important intellectual content. All authors have read and approved the final manuscript.

## Funding

This work was supported by The Joint Funds for the Innovation of Science and Technology Fujian Province to X-YC (No. 2021Y9088, Fujian).

## Conflict of interest

The authors declare that the research was conducted in the absence of any commercial or financial relationships that could be construed as a potential conflict of interest.

## Publisher’s note

All claims expressed in this article are solely those of the authors and do not necessarily represent those of their affiliated organizations, or those of the publisher, the editors and the reviewers. Any product that may be evaluated in this article, or claim that may be made by its manufacturer, is not guaranteed or endorsed by the publisher.
